# Vasoinhibins regulate the inner and outer blood-retinal barrier and limit retinal oxidative stress

**DOI:** 10.3389/fncel.2014.00333

**Published:** 2014-10-20

**Authors:** David Arredondo Zamarripa, Nundehui Díaz-Lezama, Rodrigo Meléndez García, Jesús Chávez Balderas, Norma Adán, Maria G. Ledesma-Colunga, Edith Arnold, Carmen Clapp, Stéphanie Thebault

**Affiliations:** Departamento de Neurobiología Celular y Molecular, Instituto de Neurobiología, Universidad Nacional Autónoma de MéxicoQuerétaro, México

**Keywords:** vasoinhibins, 16K prolactin, blood-retina barrier, retinal pigment epithelium, nitric oxide, reactive oxygen species, oxidative stress, diabetes

## Abstract

Vasoinhibins are prolactin fragments present in the retina, where they have been shown to prevent the hypervasopermeability associated with diabetes. Enhanced bradykinin (BK) production contributes to the increased transport through the blood-retina barrier (BRB) in diabetes. Here, we studied if vasoinhibins regulate BRB permeability by targeting the vascular endothelium and retinal pigment epithelium (RPE) components of this barrier. Intravitreal injection of BK in male rats increased BRB permeability. Vasoinhibins prevented this effect, as did the B2 receptor antagonist Hoe-140. BK induced a transient decrease in mouse retinal and brain capillary endothelial monolayer resistance that was blocked by vasoinhibins. Both vasoinhibins and the nitric oxide (NO) synthase inhibitor L-NAME, but not the antioxidant N-acetyl cysteine (NAC), blocked the transient decrease in bovine umbilical vein endothelial cell (BUVEC) monolayer resistance induced by BK; this block was reversed by the NO donor DETANONOate. Vasoinhibins also prevented the BK-induced actin cytoskeleton redistribution, as did L-NAME. BK transiently decreased human RPE (ARPE-19) cell monolayer resistance, and this effect was blocked by vasoinhibins, L-NAME, and NAC. DETANONOate reverted the blocking effect of vasoinhibins. Similar to BK, the radical initiator Luperox induced a reduction in ARPE-19 cell monolayer resistance, which was prevented by vasoinhibins. These effects on RPE resistance coincided with actin cytoskeleton redistribution. Intravitreal injection of vasoinhibins reduced the levels of reactive oxygen species (ROS) in retinas of streptozotocin-induced diabetic rats, particularly in the RPE and capillary-containing layers. Thus, vasoinhibins reduce BRB permeability by targeting both its main inner and outer components through NO- and ROS-dependent pathways, offering potential treatment strategies against diabetic retinopathies.

## Introduction

The functional integrity of the blood-retinal barrier (BRB) is crucial for proper vision. The BRB consists of inner and outer components that are mainly formed by vascular endothelial and retinal pigment epithelial (RPE) cells, respectively (Klaassen et al., 2013). Diverse conditions, including aging and diabetes, are associated with excessive transport through the BRB (Gardner et al., 2002; Klaassen et al., 2013). In this respect, increased levels of bradykinin (BK), a hypotensive peptide that belongs to the kinin-kallikrein system (Leeb-Lundberg et al., 2005), have been detected in the vitreous of patients with diabetic retinopathy (Gao et al., 2007), and the intravitreal injection of both BK and the enzyme that produces BK from high molecular weight kininogen (prekallikrein) induce excessive permeability through the BRB causing edema in rats (Phipps et al., 2009; Clermont et al., 2011). In addition, kinin receptor antagonists prevent the increase in retinal vascular permeability due to diabetes in rats (Abdouh et al., 2008).

Kinin receptors occur in two isoforms: the B2 receptor, which is ubiquitously and constitutively expressed (Sainz et al., 2007), and the B1 receptor, which is minimally expressed under physiological conditions but up-regulated during tissue injury and inflammation (Marceau and Regoli, 2004; Leeb-Lundberg et al., 2005). BK binds preferentially to the B2 receptor (Charest-Morin et al., 2014), which has been detected in the human retina (Takeda et al., 1999). In rodents, there is functional evidence of its presence in retinal capillary endothelial (Abdouh et al., 2003) and RPE (Ma et al., 1996; Lim et al., 2008, 2009) cells. Stimulation of the B2 receptor has been shown to promote vascular permeability in endothelial cells of the blood-brain barrier (Revest et al., 1991; Doctrow et al., 1994; Easton and Abbott, 2002; Raslan et al., 2010) by increasing the production of both nitric oxide (NO) *via* Ca^2+^/CaM kinase II activation (Cai et al., 2008) and of reactive oxygen species (ROS) *via* arachidonic acid (Easton and Abbott, 2002) and NADPH oxidase activation (Fischer et al., 2005). Both NO and ROS cause cytoskeleton reorganization and subsequent tight and adherens junction reorganization (De Bock et al., 2013) that, together, control endothelial cell permeability. On the other hand, NO is known to contribute to the integrity of RPE tight junctions (Zech et al., 1998), and increased ROS production correlates with increased permeability through RPE (Miura and Roider, 2009; Qin and Rodrigues, 2010; Kim et al., 2012).

A major feature of aging- and diabetes-related retinopathies is the excessive production of NO and ROS (Zheng and Kern, 2009). Therefore, more insight into the action mechanisms of molecules that can modulate the BK pathway will contribute to retinal health. Vasoinhibins, a family of peptides originating from the proteolysis of the hormone prolactin (Clapp et al., 2006), have been demonstrated to antagonize several effects of BK, including vasorelaxation, vascular production of NO (Gonzalez et al., 2004), and endothelial cell proliferation (Thebault, 2011). Moreover, vasoinhibins prevent the excessive vasopermeability associated with diabetes (Garcia et al., 2008). In this study, we investigated whether vasoinhibins reduce the BK-induced increase in BRB permeability by targeting both the endothelial and the RPE components of this barrier. We also wished to ascertain whether NO and ROS mediate these effects. To this end, we quantified transport through the BRB using the Evans blue dye method in rats, and we used monolayers of freshly isolated mouse retinal and brain capillary endothelial cells, BUVEC and ARPE-19 to assess trans-electrical resistance (TER). We also analyzed the filamentous (F-) actin distribution and contribution of the kinin B2 and B1 receptors, NO, and ROS to the mechanism of vasoinhibin action using selective pharmacological agonists and/or inhibitors. Our data support the hypothesis that vasoinhibins regulate endothelial and RPE cell permeability; furthermore, they showed that vasoinhibins attenuate diabetes-related oxidative stress in the retina, and that NO and ROS differentially contribute to the regulation of permeability through endothelial and RPE cell monolayers.

## Materials and methods

### Reagents

The vasoinhibins used in *in vivo* experiments corresponding to the 16 kDa fragment were generated by the enzymatic cleavage of rat prolactin from mammary gland extracts as previously described (Clapp et al., 1993). Recombinant human vasoinhibins (corresponding to a 14-kDa fragment of prolactin) used in cell culture experiments were generated by site-directed mutagenesis as previously described (Galfione et al., 2003). Other compounds including BK, Nω-Nitro-L-arginine methyl ester hydrochloride (L-NAME), (Z)-1-[2-(2-aminoethyl)-*N*-(2-ammonio-ethyl)amino]diazen-1-ium-1,2-diolate (DETANONOate), N-acetylcysteine (NAC), Luperox (*tert*-butyl peroxide), the kinin B2 receptor antagonist Hoe-140, and the Evans blue dye were purchased from Sigma-Aldrich (St. Louis, MO), and the 5,59,6,69-tetrachloro-1,19,3,39 tetraethylbenzimidazolylcarbocyanine iodide JC-1 dye was from Molecular probes (Eugene, OR). The monoclonal anti-kinin B2 receptor, the polyclonal anti-kinin B1 receptor, and polyclonal anti-β-tubulin antibodies were purchased from BD Biosciences (610451), Santa Cruz Biotechnology (sc-15048), and ZYMED (22833), respectively. Secondary antibodies conjugated to alkaline phosphatase (Bio-Rad Laboratories, Hercules, CA) were used.

### Ethics statement

All experiments were approved by the Bioethics Committee of the Institute of Neurobiology (INEU/SA/CB/074) from the National University of Mexico (UNAM, clave NOM-062-ZOO-1999) in accordance with the rules and regulation of the Society for Neuroscience: Policies on the Use of Animals and Humans in Neuroscience Research. All efforts were made to minimize the number of animals used and their suffering.

### Animals

Male albino rats (Wistar, 250–350 g) were fed *ad libitum* and reared in normal cyclic light conditions (12h light: 12h dark). A group of rats received L-NAME (1.8 mM) in drinking water for 15 days. Sprague-Dawley rats were immunized with Complex Freund's Adjuvant (Adan et al., 2013). For all procedures, rats were anesthetized with ketamine/xylazine (7/3). Additional anesthesia was provided throughout the procedures as needed. Diabetes was induced with a single intraperitoneal injection of streptozotocin (60 mg/kg) in Wistar rats (Garcia et al., 2008), and animals with glucose levels greater than 250 mg/dl were used 4 weeks after diabetes induction.

### Cells

BUVEC (Cajero-Juarez et al., 2002) were grown in F12K culture medium supplemented with 10% fetal bovine serum, 50 U/ml penicillin/streptomycin, and 1% fungizone (Life Technologies, Grand Island, NY). The ARPE-19 human cell line (ATCC Number: CRL-2302) (Dunn et al., 1996) was grown in Dulbecco's Modified Eagle's Medium nutrient mixture F12 supplemented with 1% fetal bovine serum. Cell cultures were cultured at an initial density of 10^6^ cells and maintained at 37°C and 5% CO_2_.

### Primary cultures of mouse brain (MBCEC) and retinal (MRCEC) capillary endothelial cells

We isolated MBCEC and MRCEC from the mouse brain and retina using collagenase digestion and magnetic-activated cell sorting (MACS), ensuring a preparation of >90% purity. Briefly, brains (devoid of cerebellum) and retinas from five to twenty Cd-1 mice (4 weeks of age) were aseptically collected and rinsed in Hank's Balanced Salt Solution (GIBCO BRL, New York, NY) supplemented with 1% bovine serum albumin (BSA) and 1% penicillin/streptomycin, and were cut into small pieces. Tissue was digested with 0.1% collagenase type 1 for 45 min at 37°C under agitation (350 rpm). The cell-suspension was then filtered through 70-micron mesh and centrifuged at 1200 rpm for 10 min at 4°C. Supernatant from the brain-derived suspension was discarded, and the pellet was treated with 2 mL of erythrocyte lysis buffer for 5 min and centrifuged. Finally, CD31-positive cells from both brain and retinal populations were isolated by MACS as previously described (Smith et al., 2014).

### Intravitreal injections

Rats were injected intravitreously as reported (Aranda et al., 2005). The final injection volume was 5 μl. One eye was injected with BK (60 pg per eye, corresponding to 1 nM as the estimated volume of rat vitreous is 60 μl, Guo et al., 2007) and the contra-lateral eye with BK combined with vasoinhibins (1 μg, 1 μM) or with the kinin B2 receptor antagonist Hoe-140 (235 ng, 3 μM). The control groups consisted of rat eyes injected with vehicle (PBS), and the contralateral eye received vasoinhibins (1 μM) or Hoe-140 (3 μM).

### Quantification of BRB permeability

The Evans blue dye permeation assay was performed as previously described (Xu et al., 2001; Garcia et al., 2008). It is however worth mentioning that Evans blue dye was administered 2 h after intravitreous injections, and left circulation for 2 additional hours.

### Real-time PCR

*RNA was isolated and cDNA synthesized from a* 1-μg sample *as previously described* (Arnold et al., 2014). Primer sequences are documented in Table 1, and the conditions used for the PCR reactions were *as previously described* (Arnold et al., 2014). Cycle thresholds normalized to the housekeeping gene hypoxanthine-guanine phosphoribosyltransferase (HPRT) were used to calculate the mRNA levels of interest.

**Table 1:** **Details of primer sequences**.

**mRNA**	**NCBI accession number**	**Forward sequence**	**Reverse sequence**	**Primer efficiency (%)**
Hprt	NM_012583.2	TTGCTGACCTGCTGGATTAC	GTTGAGAGATCATCTCCACC	100.8
B2R	NM_173100.2	CCCTTCCTCTGGGTCCTCTT	CAGAACACGCTGAGGACAAAGA	93.5

### Western-blot

Rat retinas and knees were homogenized in 0.5% Nonidet P-40, 0.1% SDS, 50 mM Tris, 150 mM NaCl, 1 mM phenylmethylsulnonyl fluoride, and 1 mM aprotinin (pH 7) and centrifuged (10,000 *g* for 10 min). Supernatant proteins (40 μg) were resolved by 7.5 or 10% SDS-polyacrylamide gels under reducing conditions, transferred to nitrocellulose membranes, and probed with an anti-kinin B2 or B1 receptor (1:500 and 1:50 dilution, respectively) and anti-β-tubulin (1:1000 dilution) antibodies. Primary antibodies were revealed using secondary antibodies conjugated to alkaline phosphatase. Optical density values were determined using the Quantity One, 1-D analysis software (Bio-Rad, Hercules, CA).

### F-actin staining

Cells were grown on matrigel-coated coverslips until confluence. After treatment, cells were fixed with 4% paraformaldehyde for 10 min at room temperature. Then cells were washed twice with PBS and permeabilized with 0.1% Triton X-100 in PBS for 5 min. Changes in F-actin structures were detected by incubating the cells for 20 min at room temperature with a 1:100 dilution of TRITC-labeled phalloidin (Molecular Probes). Coverslips were then washed twice with PBS and mounted in VECTASHIELD® mounting medium with DAPI. Images were obtained using a LSM 510 confocal laser scanning microscope (Carl Zeiss, Jena, Germany).

### Measurement of TER

BUVEC, MRCEC, MBCEC, and ARPE-19 cells were grown on 1.12-cm^2^ Transwell clear polyester membrane inserts (Corning Inc., Corning, NY) with pore sizes of 8.0 μm for BUVEC and 0.4 μm for other cells, and monitored until stabilization of TER (average of 180 to 200 Ω.cm^2^) using the EVOM2 Epithelial Voltohmmeter (World Precision Instrument). TER was then measured over a 90-min period from each insert unless another time period is specified. TER values were expressed as percent of control (complete medium) at time 0.

### Assessment of mitochondrial ROS production using JC-1 dye

Hyperpolarization of the mitochondrial membrane potential results in increased ROS generation (Teshima et al., 2014). The JC-1 dye is freely permeable to cells and undergoes reversible transformation from a monomer (emitting at ~530 nm (green) in response to a 535-nm excitation) to an aggregate form (emitting red fluorescence at ~590 nm (red) in response to a 485-nm excitation) when it binds to hyperpolarized mitochondrial membranes (Cossarizza et al., 1993). A decrease in the aggregate fluorescence (red; 590) is indicative of depolarization, whereas an increase indicates hyperpolarization. BUVEC (1 × 10^5^ cells/well) and ARPE-19 (1.5 × 10^5^ cells/well) cells in 96-well plates were treated as indicated in Figures **4E, 6D** diagrams. The cells were then washed and incubated in the dark with 10 μg/mL JC-1 dye at 37°C for 10 min. The mitochondrial membrane potential was measured using a bioplate reader (Victor^3^ V [Perkin Elmer]; green fluorescence, 530 nm; red fluorescence, 570 nm).

### Measurement of superoxide using the victor^3^ V microplate reader

Superoxide levels were monitored according to the Manufacturer's instructions (The Enzo Life Sciences' Total ROS/Superoxide detection kit; ENZ-51010).

### Fluorescent detection of intracellular ROS

The *in vivo* production of ROS in the retina was assessed using dihydroethidium (DHE) staining. First, eyes were fixed in 4% PFA for 4 h and then placed in 15 % sucrose for 1 day. Eyes were frozen (Tissue-Tek; Sakura Finetek, Torrance, CA), and 20-μm cryostat sections were mounted on gelatin-coated slides. In a dark chamber, retinal sections were loaded with 5 μM DHE for 30 min at room temperature and then washed thrice in PBS. Sections were mounted on glass slides with VECTASHIELD® mounting medium with DAPI, and the slides were analyzed without delay using a LSM 510 confocal laser-scanning microscope. Images were obtained using an Apo-406 objective. The same acquisition settings were used for all experiments to allow direct comparison of retinal explants. Digital images were processed using free ImageJ software (Rasband, W.S., ImageJ; U.S. NIH, Bethesda). Only cropping of images was performed; there was no adjustment of brightness. The mean fluorescence intensity ratio of each retinal layer was determined in 6 different regions of interest (same size), distributed equally over the full layer, from one retinal section per time point (each of which was representative of 3 experiments). The ratio between PBS (numerator) and vasoinhibins, time-matched control retinas was calculated by 2 single-blind, independent observers to determine superoxide anion production in specific treatment groups.

### Statistical analysis

All results were replicated in three or more independent experiments. Data were presented as mean ± s.e.m.; all data showed normal distribution or equal variance according to Kolmogorov-Smirnov and Levene's tests, respectively. Statistical differences among groups were determined by One-Way ANOVA followed by Bonferroni's *post-hoc* comparison test (Sigma Stat 7.0, Systat Software Inc., San Jose, CA). Differences in means with *P* < 0.05 were considered statistically significant.

## Results

### Vasoinhibins prevent BK-induced increase in BRB permeability similarly to a kinin B2 receptor antagonist

**Figure 1:**
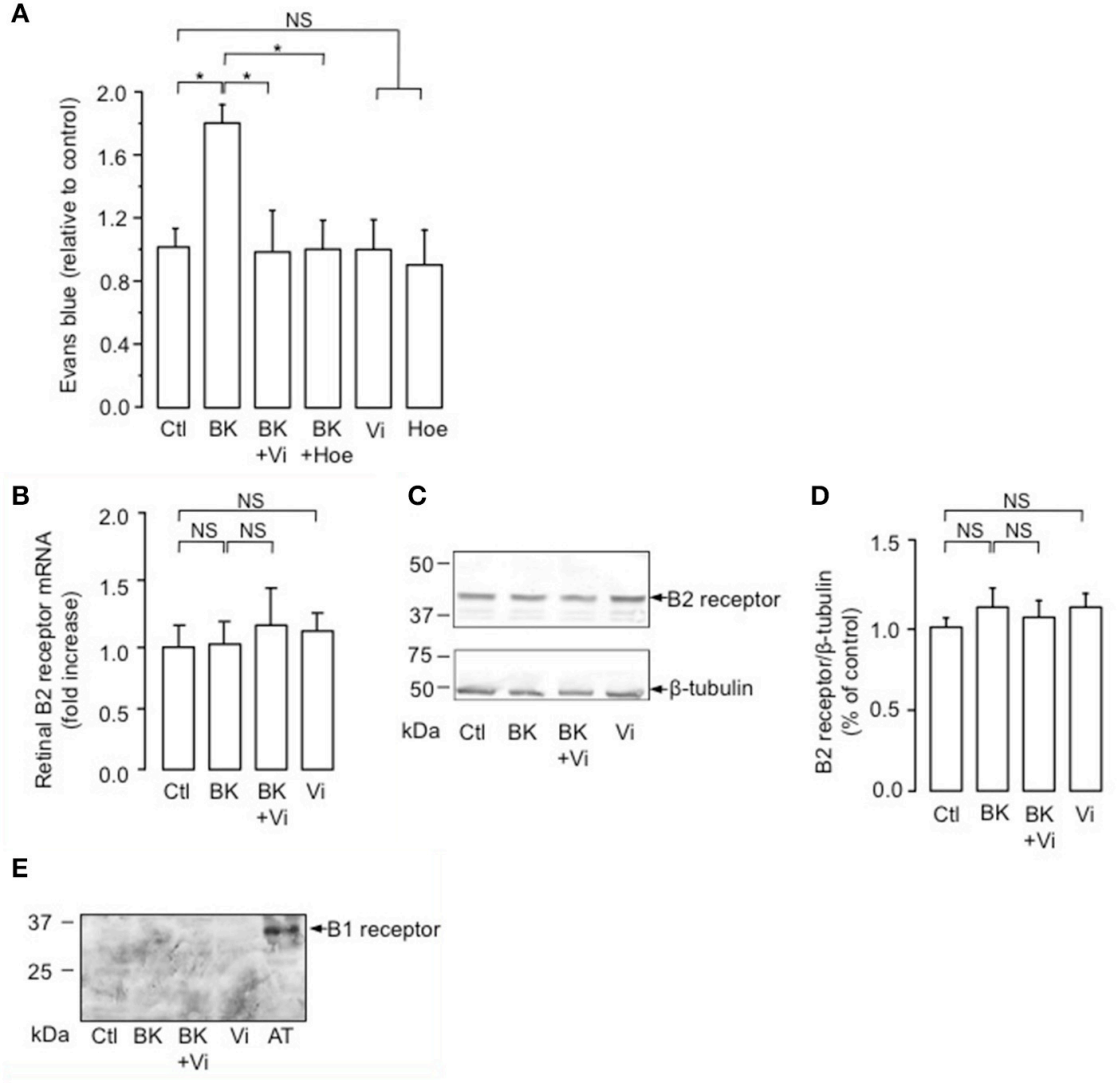
**Vasoinhibins prevent BK-induced increase in BRB permeability similarly to a kinin B2 receptor antagonist**. **(A)** Evaluation of the Evans blue dye content in retinas of rats intravitreously injected 4 h earlier with PBS (Ctl), BK (1 nM), BK combined with either vasoinhibins (Vi, 1 μM) or Hoe-140 (Hoe, 3 μM), Vi alone, or Hoe alone. Values are mean ± s.e.m. normalized to the control (*n* = 8–16 per group; ^*^P < 0.05). **(B–D)** Retinas of rats that were intravitreously injected 4 h earlier with PBS, BK, Vi, or BK combined with Vi were analyzed for kinin B2 receptor mRNA (B, mean ± s.e.m. from 5 independent experiments) and protein **(C)**. Total β-tubulin served as loading control. **(D)** Quantification of kinin B2 receptor by densitometry normalized to total β-tubulin. Values correspond to the mean ± s.e.m. from 3 independent experiments. **(E)** Retinas of rats that were intravitreously injected 4 h earlier with PBS, BK, Vi, or BK combined with Vi were analyzed for kinin B1 receptor protein. Total knee extract from rats with Freund's adjuvant-induced arthritis (AT, arthritic tissue) was used as a positive control for B1 receptor expression. NS, not significant.

The intravitreal injection of BK induced a 1.8-fold increase in the basal transport through the BRB compared to the PBS-injected eyes (Ctl) (Figure 1A). When coinjected with BK, vasoinhibins prevented the BK-induced increase in BRB permeability in a manner similar to that of the competitive kinin B2 receptor antagonist Hoe-140 (Figure 1A). Alone, vasoinhibins or Hoe-140 did not modify the transport through the BRB. Signaling through the kinin B2 receptor has been shown to be primarily controlled by short-term mechanisms including both receptor desensitization (Mathis et al., 1996) and internalization (Munoz and Leeb-Lundberg, 1992; Munoz et al., 1993), but it can also be regulated by changes in expression levels of the receptor (Nostramo et al., 2013). Thus, retinas of animals that were intravitreally injected with BK, vasoinhibins, or both were analyzed for B2 receptor mRNA and protein levels by real-time PCR and Western-blot, respectively. The retinal levels of B2 receptor mRNA (Figure 1B) and protein (Figure 1C) were not different between PBS-, BK-, Vi-, and (BK + Vi)-injected eyes. Figure 1D shows quantification of B2 receptor after normalization for the amount of β-tubulin on the gel. Treatment with BK has been shown to induce the kinin B1 receptor (Phagoo et al., 1999). However, in addition to being absent in rat retina under physiological conditions, the B1 receptor is also absent after BK injection, combined or not with vasoinhibins (Figure 1E). Total knee extract from rats subjected to the adjuvant model of inflammatory arthritis for 21 days (Adan et al., 2013) was used as a positive control for B1 receptor expression. Notably, the band detected in arthritic tissue (AT) with the polyclonal anti-B1 receptor antibody migrates at an apparent molecular weight of 35 kDa, the molecular mass of the B1 receptor (see http://datasheets.scbt.com/sc-15048.pdf), thus validating the efficacy of the Western blot detection. These data indicate that vasoinhibins mitigate the BK-mediated increase in BRB permeability, that this results from kinin B2, but not B1, receptor activation, and that vasoinhibins do not change the amount of B2 receptor.

### Vasoinhibins block the reduction of trans-electrical resistance (TER) induced by BK in MRCEC, MBCEC, and BUVEC monolayers

**Figure 2:**
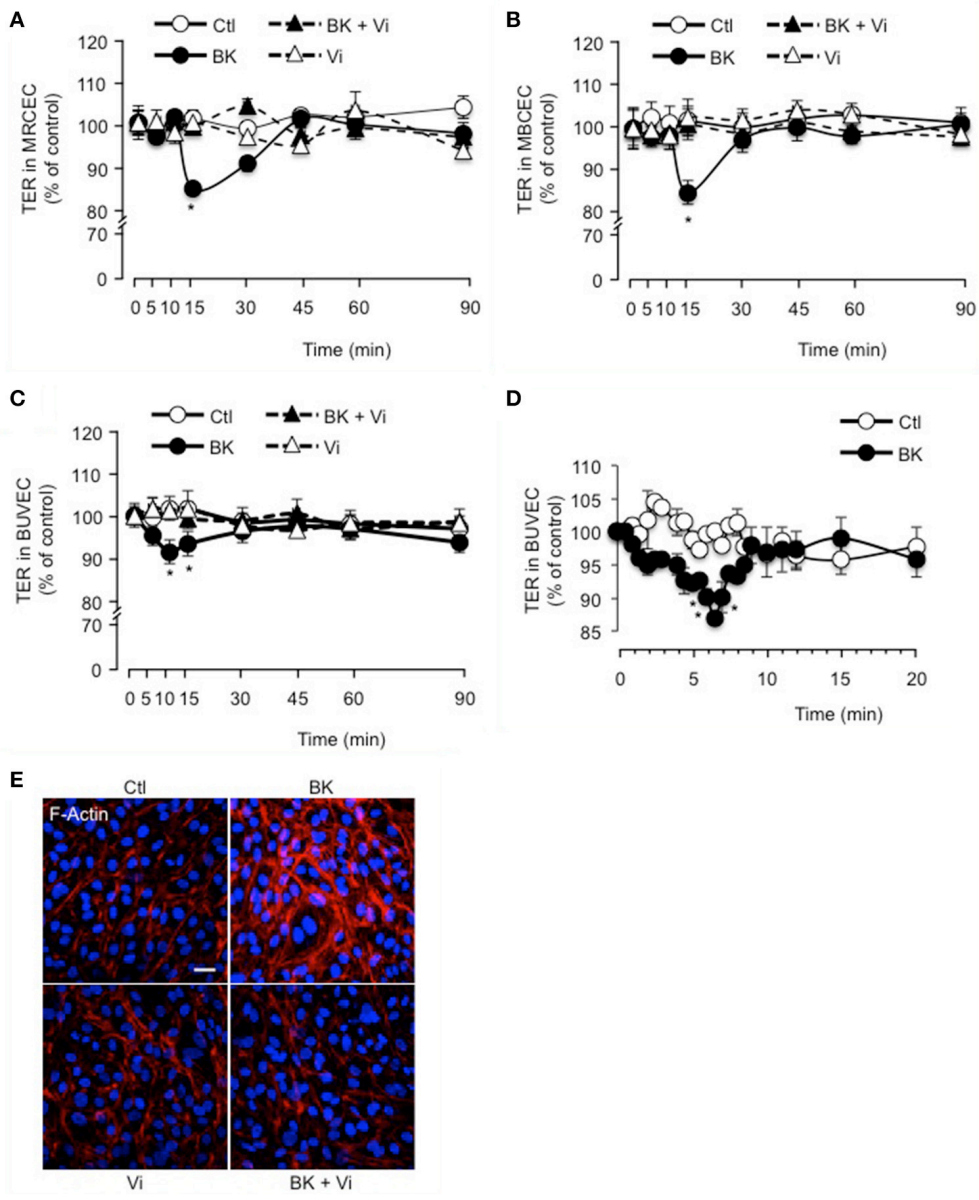
**Vasoinhibins block BK-induced reduction of transendothelial resistance and morphological changes in actin cytoskeleton**. Time course of trans-electrical resistance (TER) in MRCEC **(A)** and MBCEC **(B)** monolayers cultured in complete medium (Ctl) with or without 10 μM BK and 10 nM vasoinhibins (Vi). **(C)** TER in BUVEC monolayers cultured in complete medium (Ctl) with or without 10 μM BK and 10 nM Vi. **(D)** Expanded early time values of experiment **(C)**. In **(A–D)**, values are mean ± s.e.m. from 3 independent experiments normalized to the control; ^*^P < 0.05 vs. Ctl. MBCEC and MRCEC were cultured on inserts with pore sizes of 0.4 μm while BUVEC cells were cultured on inserts with pore sizes of 8.0 μm. **(E)** BUVEC were cultured in complete medium (Ctl) with or without 10 μM BK and 10 nM Vi for 15 min and then actin cytoskeleton (F-actin) distribution was determined using rhodamine-phalloidin. Representative fields are shown. Scale bar, 10 μm.

The inner BRB refers to the layer of capillaries in the inner retina with very low permeability, due to the interactions between vascular endothelial cells and surrounding cells (pericytes and macroglia) (Klaassen et al., 2013). Freshly isolated mouse retinal (Figure 2A) and brain (Figure 2B) capillary endothelial cells showed stable resistance over time. BK induced a transient decrease in TER that was maximal at 15 min and was fully prevented by vasoinhibins (Figures 2A,B). Vasoinhibins alone had no effect (Figures 2A,B). Monolayers of BUVEC that express B2 receptors (Wohlfart et al., 1997), also showed stable resistance over time (Figure 2C). BK induced a transient decrease in TER that was maximal at 10 min (Figures 2C,D) and was fully prevented by vasoinhibins (Figure 2C). Vasoinhibins alone had no effect (Figure 2C). The cytoskeleton is composed of actin microfilaments, which are critical for endothelial cell permeability (Dudek and Garcia, 2001). Treatment with BK induced F-actin redistribution and stress fiber formation in BUVEC (Figure 2E). These effects were blocked by vasoinhibins, and vasoinhibins had no effect alone (Figure 2E).

### Vasoinhibins block the BK-induced decrease in BUVEC resistance and actin cytoskeleton redistribution by acting upstream of NO production

**Figure 3:**
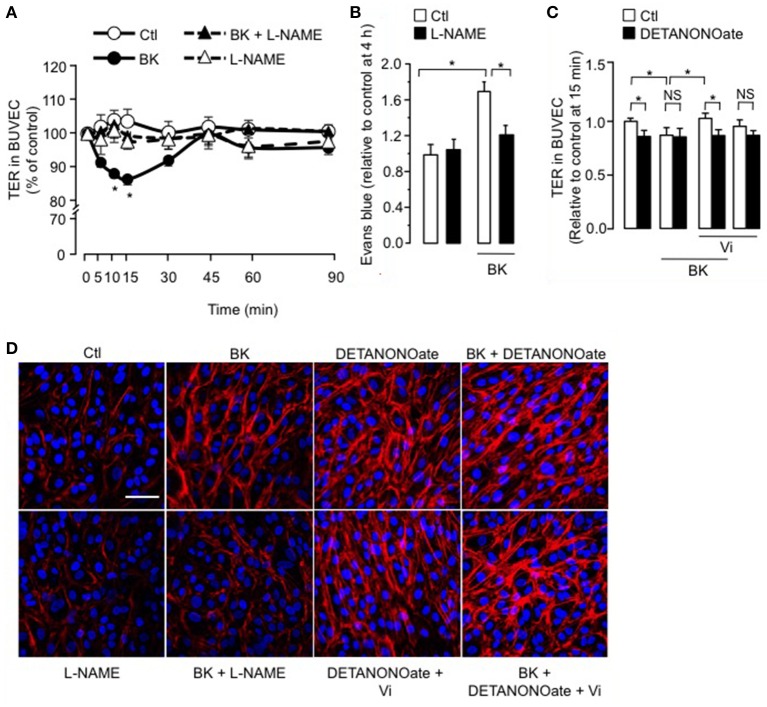
**The inhibitory effect of vasoinhibins on BK-induced reduction of transendothelial resistance and actin cytoskeleton rearrangement depends on NO. (A)** Time course of TER in BUVEC monolayers cultured in complete medium (Ctl) with or without 10 μM BK and the NO synthase inhibitor L-NAME (10 mM). Values are mean ± s.e.m. from 3 independent experiments normalized to the control. ^*^P < 0.05 vs. Ctl. **(B)** Rats were treated with L-NAME (1.8 mM) administered in the drinking water for 15 days, then their retinas were intravitreously injected with PBS (Ctl) or BK (1 nM), and evaluated 2 h later by the Evans blue dye assay. Values are mean ± s.e.m. normalized to control. ^*^P < 0.05 from 8 to 16 independent observations. **(C)** Quantification of peak TER values (15 min after treatment start) in BUVEC monolayers cultured in complete medium (Ctl) with or without 10 μM BK and 10 nM Vi in the absence (white bars) or presence (black bars) of the NO donor DETANONOate (10 μM). ^*^P < 0.05 from 3 independent experiments. NS, not significant. BUVEC were cultured on inserts with pore sizes of 8.0 μm. **(D)** BUVEC were cultured in complete medium (Ctl) with or without 10 μM BK and 10 mM L-NAME, and with the NO donor DETANONOate (10 μM) in the presence and in the absence of BK and Vi (10 nM) for 15 min, and then actin cytoskeleton (F-actin) distribution was determined using rhodamine-phalloidin. Representative fields are shown. Scale bar, 10 μm.

We observed that coadministration of the NOS inhibitor L-NAME (Rees et al., 1989) eliminated the BK-induced decrease in TER, but L-NAME alone had no effect (Figure 3A). Administration of L-NAME (1.8 mM) in the rats' drinking water for 15 days also counteracted BK action on BRB permeability (Figure 3B), mimicking the vasoinhibin effect (Figure 1A). In support of NO being essential for BK effects, the NO donor DETANONOate mimicked the BK effect on TER, and coadministration of DETANONOate did not enhance BK action (Figure 3C). We then asked whether the NO donor DETANONOate reverts the action of vasoinhibins in the presence of BK. Exogenous NO prevented vasoinhibin-mediated inhibition of BK-induced reduction of TER in BUVEC (Figure 3C). In addition, L-NAME prevented BK-induced redistribution of F-actin and stress fiber formation, but had no effect alone (Figure 3D). Also, DETANONOate induced F-actin redistribution and stress fiber formation similarly to BK, and coadministration of DETANONOate did not enhance BK action (Figure 3D). In this context, vasoinhibins were not able to block DETANONOate-induced F-actin rearrangement, and exogenous NO prevented vasoinhibin-mediated inhibition of BK-induced F-actin redistribution in BUVEC (Figure 3D). All together, these data show that NO mediates the BK-induced increase in endothelial cell monolayer permeability and actin cytoskeleton redistribution, and that vasoinhibins block these actions by acting upstream of NO production, as previously reported (Gonzalez et al., 2004; Thebault, 2011).

### The vasoinhibin-mediated inhibition of TER reduction and actin cytoskeleton redistribution induced by BK is independent of intracellular ROS production

**Figure 4:**
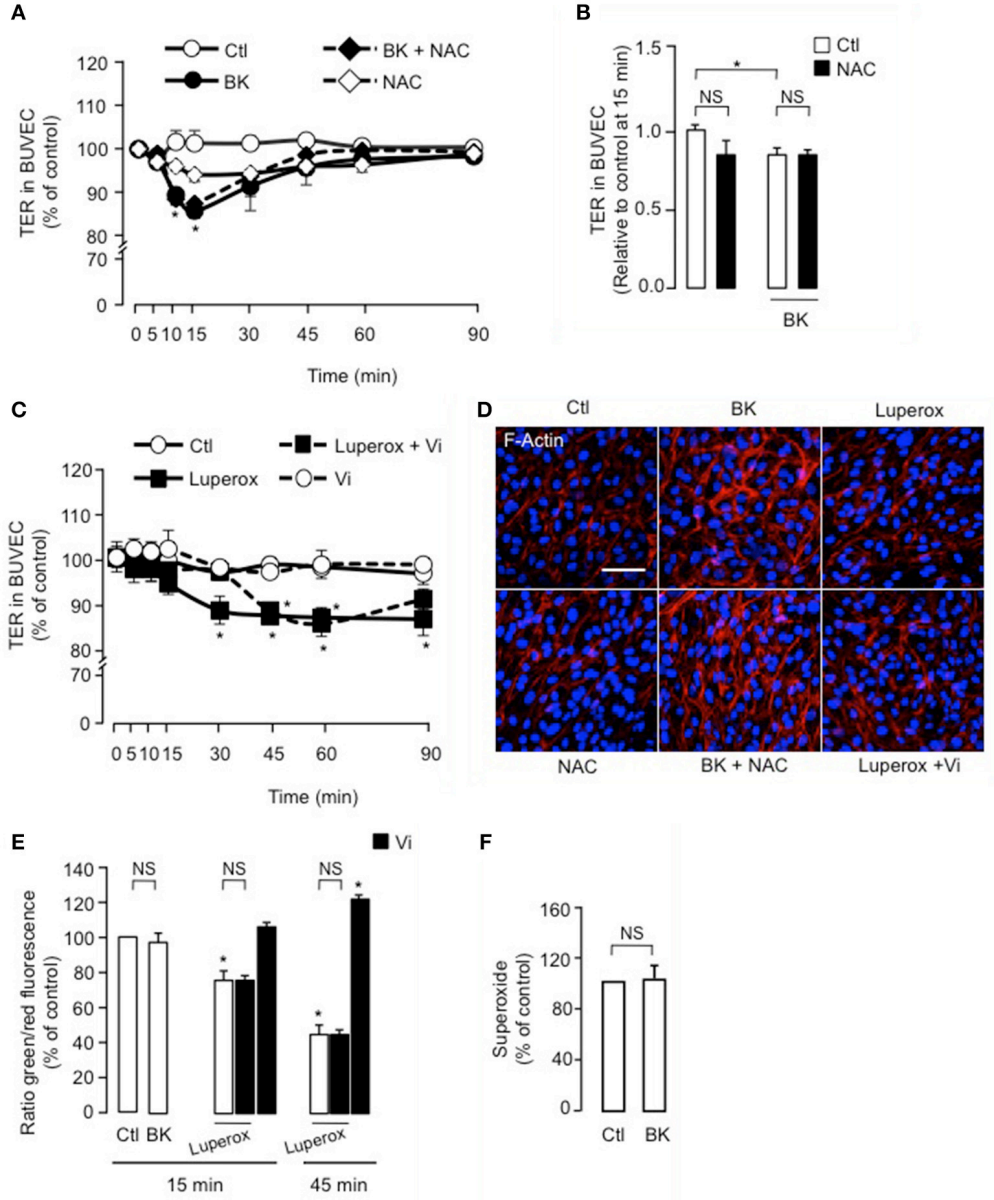
**ROS do not participate in the vasoinhibin-mediated inhibition of transendothelial resistance reduction and actin cytoskeleton rearrangement induced by BK**. **(A,C)** Time course of trans-electrical resistance (TER) in BUVEC monolayers cultured in complete medium (Ctl) with or without 10 μM BK and the antioxidant N-acetyl cysteine (NAC, 10 mM) or with or without 500 μM Luperox and vasoinhibins (Vi, 10 nM). BUVEC were cultured on inserts with pore sizes of 8.0 μm. **(B)** Corresponding quantification of TER values, 10 min after treatment initiation. Values correspond to the mean ± s.e.m. from 3 independent experiments. ^*^P < 0.05. NS, not significant. **(D)** BUVEC were cultured in complete medium (Ctl) with or without 10 μM BK and 10 mM NAC or with or without 500 μM Luperox and Vi (10 nM) for 15 min, and then actin cytoskeleton (F-actin) distribution was determined using rhodamine-phalloidin. Representative fields are shown. Scale bar, 10 μm. **(E)** Mitochondrial membrane potential changes using JC-1 dye in BUVEC monolayers cultured in complete medium (Ctl) with or without 10 μM BK or 500 μM Luperox and 10 nM Vi. Values correspond to the mean ± s.e.m. from 3 independent experiments. ^*^P < 0.05. NS, not significant. **(F)** Cytosolic levels of superoxide in BUVEC monolayers cultured in complete medium (Ctl) with or without 10 μM BK. Values correspond to the mean ± s.e.m. of 8 repeats per condition from 3 independent experiments.

Evidence suggests that increased retinal vascular permeability in response to agonists such as BK is associated with increased ROS load (Wohlfart et al., 1997; Fong et al., 2010), which can be reduced inside the cell by free radical scavengers like NAC (Zavodnik et al., 2013). We observed that NAC did not block BK-induced reduction of TER in BUVEC (Figure 4A). Figure 4B shows the quantification of TER values at 15 min, when the BK effect was maximal. On the other hand, the free radical initiator Luperox (Zavodnik et al., 2013) did reduce TER in BUVEC after 30 min, an effect that persisted throughout the 90-min recording period (Figure 4C). Vasoinhibins delayed the action of Luperox by 15 min, without affecting the magnitude of its effect (Figure 4C). Furthermore, the BK-induced F-actin redistribution and stress fiber formation was not prevented by the antioxidant NAC, and NAC alone had no effect (Figure 4D). Luperox did not significantly affect F-actin distribution, nor did it when co-administered with vasoinhibins (Figure 4D). Further, we assessed the levels of ROS generated by mitochondrial oxidative phosphorylation, using the JC-1 probe. Levels of ROS were assessed in BUVEC monolayers treated with BK for 15 min, when its decrease of TER is maximal. BK did not modify intracellular ROS levels compared with untreated BUVEC monolayers (Figure 4E). A 15- and 45-min exposure of BUVEC to Luperox decreased the 530/590 optical density ratio, indicating increased levels of intracellular ROS (Figure 4E). Concomittant administration of vasoinhibins did not prevent the Luperox-induced production of ROS. While ROS levels were not modified after a 15-min incubation with vasoinhibins alone, they were reduced when the incubation with vasoinhibins was extended to 45 min (Figure 4E). In addition, ROS can be generated from oxidoreductase enzymes and metal-catalyzed oxidation. However, BK did not modify cytosolic levels of superoxide in BUVEC (Figure 4F). The observations that BK and Luperox acted with different kinetics, that NAC did not block the BK effect, and that BK does not modify the levels of ROS generated as byproducts during mitochondrial electron transport or as intermediates of metal-catalyzed oxidation reactions, indicate that ROS do not contribute to BK action. Our data also show that vasoinhibins delay the ROS effect on transendothelial resistance and that the vasoinhibins *per se* reduce the intraendothelial levels of ROS.

### BK induces a transient reduction of ARPE-19 cell monolayer resistance and an actin cytoskeleton redistribution that involve NO, and vasoinhibins prevent these effects

**Figure 5:**
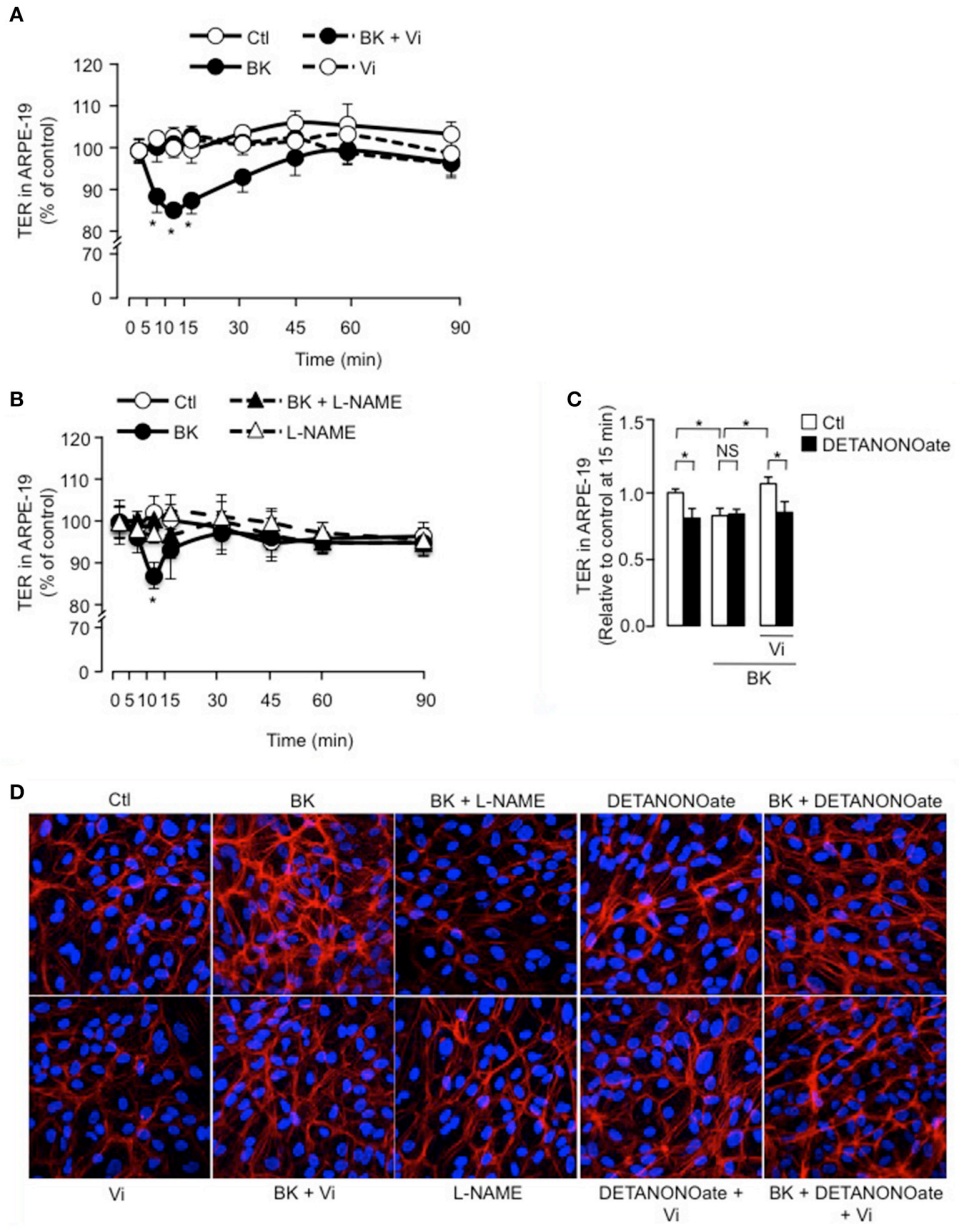
**BK transiently reduces ARPE-19 cell monolayer resistance and induces actin cytoskeleton rearrangement through NO, and vasoinhibins prevent these effects**. **(A,B)** Time course of trans-electrical resistance (TER) in ARPE-19 monolayers cultured in complete medium (Ctl) with or without 10 μM BK and vasoinhibins (Vi, 10 nM) or L-NAME (10 mM). Values correspond to the mean ± s.e.m. from 3 independent experiments. ^*^P < 0.05 vs. Ctl. **(C)** Quantification of peak TER values (15 min after treatment start) in ARPE-19 monolayers cultured in complete medium (Ctl) with or without 10 μM BK and 10 nM Vi in the absence (white bars) or presence (black bars) of the NO donor DETANONOate (10 μM). ^*^P < 0.05 from 3 independent experiments. NS, not significant. ARPE-19 cells were cultured on inserts with pore sizes of 0.4 μm. **(D)** ARPE-19 cells were cultured in complete medium (Ctl) with or without 10 μM BK and vasoinhibins (Vi, 10 nM) or L-NAME (10 mM) or the NO donor DETANONOate (10 μM) for 15 min, and then actin cytoskeleton (F-actin) distribution was determined using rhodamine-phalloidin. Representative fields are shown. Scale bar, 10 μm.

We then tested if vasoinhibins also target the RPE component of BRB using the ARPE-19 cell line that expresses the B2 receptor (Yasuyoshi et al., 2000; Lim et al., 2008). In control conditions, the ARPE-19 monolayers showed stable resistance over time (Figure 5A). BK induced a transient decrease in TER of ARPE-19 monolayers; this reduction was similar in magnitude (15 ± 1 vs. 9 ± 2 %) and time-course (maximum at 10–15 min) to the one observed in BUVEC monolayers. Vasoinhibins prevented the BK-induced decrease in ARPE-19 monolayer resistance, but they had no effect alone (Figure 5A). Similarly, blocking NO production with L-NAME abrogated the action of BK (Figure 5B). Addition of exogenous NO (DETANONOate) reduced TER in ARPE-19 monolayers, mimicking the BK effect, and it prevented vasoinhibins from blocking the BK-induced reduction of TER (Figure 5C). Concomittantly, treatment with BK induced F-actin redistribution in ARPE-19, an effect that was blocked by vasoinhibins (Figure 5D), which had no effect alone (Figure 5D). L-NAME prevented BK-induced F-actin redistribution and had no effect alone (Figure 5D). The NO donor DETANONOate also promoted F-actin redistribution; co-administration of BK and DETANONOate did not further promote F-actin rearrangement but in the presence of BK and DETANONOate, vasoinhibins did not inhibit the F-actin redistribution (Figure 5D).

### Intracellular ROS contribute to the inhibition of BK-induced decrease of ARPE-19 resistance and actin cytoskeleton redistribution by vasoinhibins

**Figure 6:**
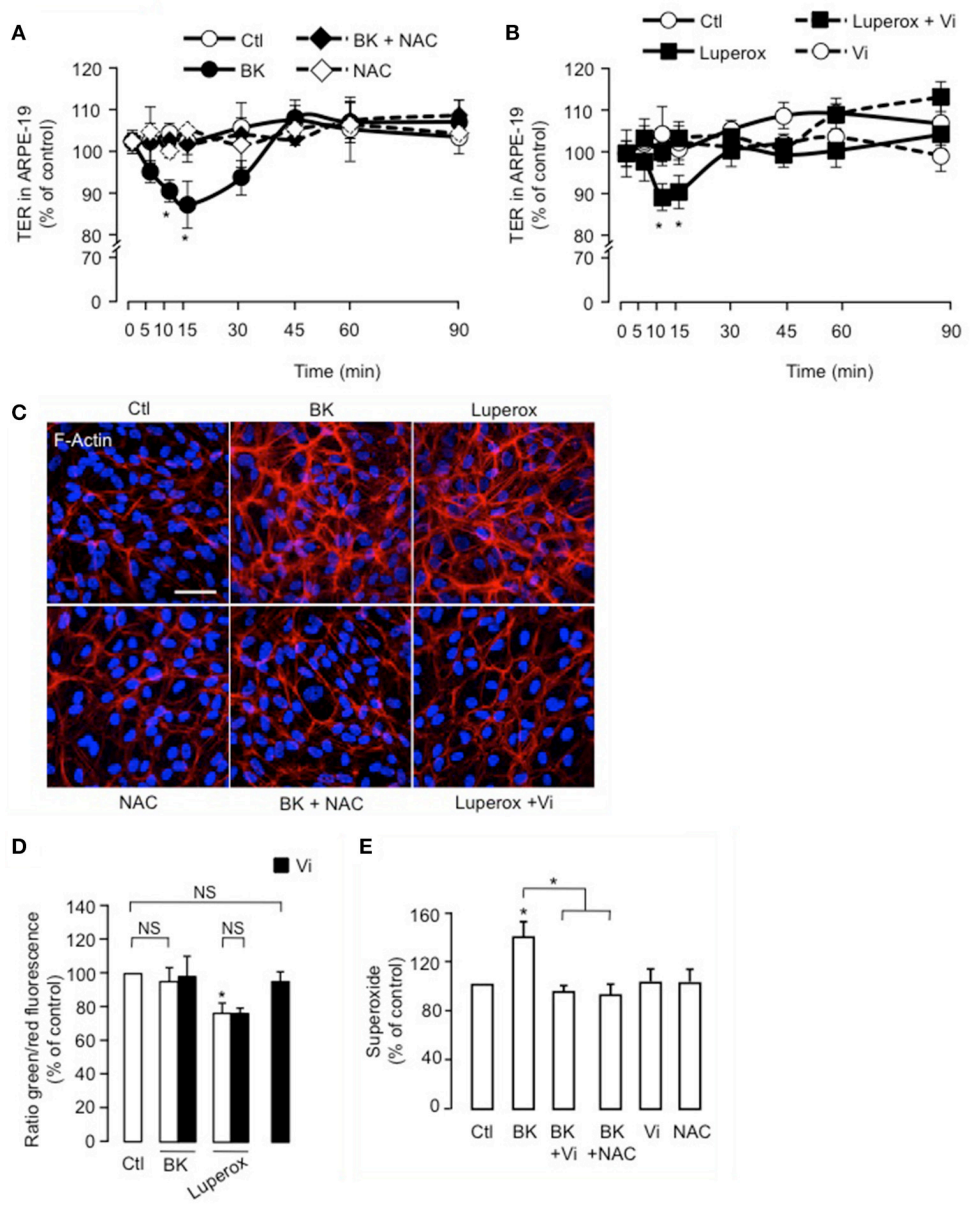
**ROS contribute to the inhibition of BK-induced decrease of ARPE-19 resistance and actin cytoskeleton rearrangement by vasoinhibins**. **(A,B)** Time course of trans-electrical resistance (TER) in ARPE-19 monolayers cultured in complete medium (Ctl) with or without 10 μM BK and N-acetyl cysteine (NAC, 10 mM) or with or without 500 μM Luperox and vasoinhibins (Vi, 10 nM). Values correspond to the mean ± s.e.m. from 3 independent experiments. ^*^P < 0.05 vs. Ctl. ARPE-19 cells were cultured on inserts with pore sizes of 0.4 μm. **(C)** ARPE-19 cells were cultured in complete medium (Ctl) with or without 10 μM BK and NAC (10 mM) or with or without 500 μM Luperox and Vi (10 nM) for 15 min, and then actin cytoskeleton (F-actin) distribution was determined using rhodamine-phalloidin. Representative fields are shown. Scale bar, 10 μm. **(D)** Mitochondrial membrane potential changes using JC-1 dye in ARPE-19 monolayers cultured in complete medium (Ctl) with or without 10 μM BK or 500 μM Luperox and Vi (10 nM). Values correspond to the mean ± s.e.m. from 3 independent experiments. **(E)** Cytosolic levels of superoxide in ARPE-19 monolayers cultured in complete medium (Ctl) with or without 10 μM BK and NAC (10 mM) or with or without 10 μM BK and Vi (10 nM). Values correspond to the mean ± s.e.m. of 4–15 repeats per condition from 3 independent experiments. ^*^P < 0.05 vs. Ctl. NS, not significant.

The antioxidant NAC blocked BK-induced reduction of TER in ARPE-19 (Figure 6A), but alone, NAC had no effect. Also, Luperox induced a transient decrease in TER that was prevented by vasoinhibins (Figure 6B). Both the magnitude (15 ± 1 vs. 9 ± 2%) and kinetics (maximum at 10 vs. 15 min) of the Luperox effect were similar to those of BK. NAC had no effect alone but blocked the BK-induced F-actin redistribution (Figure 6C). Similarly to BK, Luperox induced F-actin redistribution, an effect that was inhibited by vasoinhibins (Figure 6C). Intracellular levels of ROS were assessed in the presence and absence of vasoinhibins with BK or Luperox for 15 min, when their effects on TER were maximal. BK did not modify ROS levels in ARPE-19, while Luperox increased them (Figure 6D). Alone or combined with BK or Luperox, vasoinhibins had no effect (Figure 6D). In contrast, BK increased cytosolic levels of superoxide in ARPE-19 at 15 min, and both NAC and vasoinhibins blocked this increase (Figure 6E).

### Vasoinhibins reduce the levels of ROS in the retina of streptozotocin-induced diabetic rats

**Figure 7:**
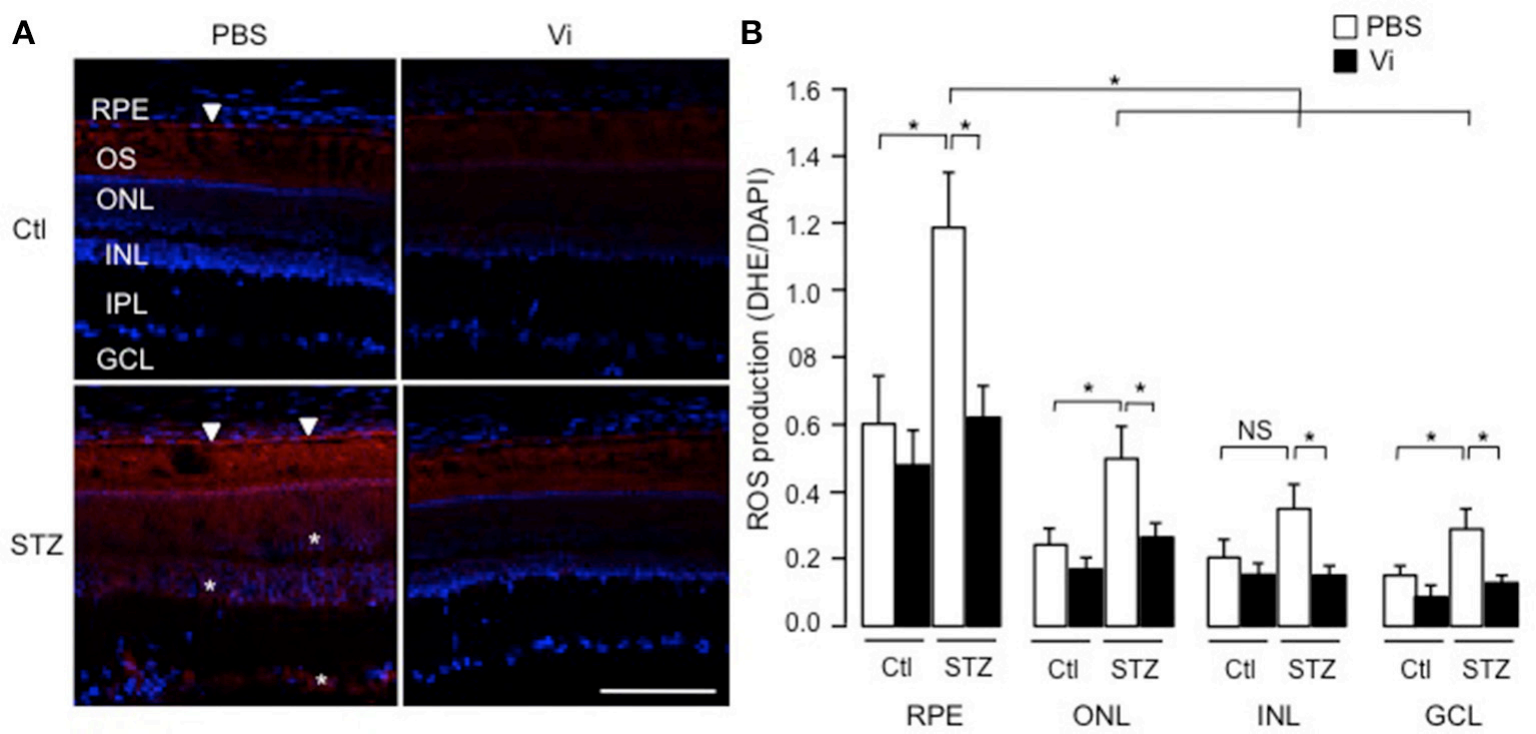
**Vasoinhibins reduce the retinal levels of ROS in streptozotocin-induced diabetic rats**. **(A)** Representative images of superoxide anion production stained with dihydroethidium (DHE) on retina sections from control (Ctl) rats intravitreously injected with PBS or vasoinhibins (Vi, 1 μM) for 24 h and from streptozotocin (STZ)-induced diabetic rats intravitreously injected with PBS or Vi 24 h before the end of the 4 weeks of diabetes. Scale bar is 100 μm. **(B)** Fluorescence intensity of superoxide anion quantified as mean number of pixels positive for DHE staining normalized to the mean number of pixels positive for DAPI staining in the retinal pigment epithelium (RPE), outer nuclear (ONL), inner nuclear (INL), inner plexiform (IPL), and ganglion cell (GCL) layers. Data are mean ± s.e.m. of values obtained from 3 rats in each group. ^*^P < 0.05. NS, not significant.

To assess the physiological relevance of our findings, we analyzed the production of superoxide anion in retinas of rats that were rendered diabetic (Pouliot et al., 2012). Figure 7A shows representative images of retinas stained with the oxidative fluorescent dye DHE (red) and the DNA stain DAPI (blue), and quantification using the mean pixel fluorescence intensity ratio of DHE/DAPI for each nuclear layer of the retina is provided in Figure 7B. In all conditions, the outer segments of photoreceptors were positive for DHE staining due to non-specific autofluorescence (Ling-Ling Zhao et al., 2009). In non-diabetic retinas, the RPE produced more superoxide anion than other layers (shown with an arrow head). After 4 weeks of diabetes, superoxide anion production was enhanced in the RPE (indicated with arrow heads in micrograph), outer nuclear and ganglion cell layers (indicated with asterisks in micrograph), as previously reported (Pouliot et al., 2011). Intravitreal injection of vasoinhibins abolished the diabetes effect on levels of superoxide anions in the RPE and the three nuclear layers of the retina. Alone, vasoinhibins had no effect on basal production of superoxide anions in the retina.

## Discussion

BRB capillary endothelial and RPE cells exhibit regulated transcytotic activity and a tight junctional network that, aided by the cytoskeleton, restricts paracellular permeability. Both trans- and paracellular pathways are the subject of extensive research as they relate to retinal degeneration, edema and inflammation. Notably, increased permeability is a prominent feature of oxidative insult to the retina. Vasoinhibins potently inhibit the increase in retinal vasopermeability associated with diabetes (Garcia et al., 2008), but their effect on the RPE component of the BRB and on oxidant-related mechanisms in the retina is unknown. Here, we show that vasoinhibins regulate BRB permeability by targeting both endothelial and RPE cells, and by limiting excessive oxidative stress in the diabetic retina. BK is an important disruptor of the BRB in diabetes that can be counteracted by vasoinhibins. BK alters not only the vascular but also the RPE compartment. After confirming that NO mediates the BK-induced decrease in endothelial monolayer resistance, we show for the first time that ROS are not involved in this effect but that both NO and ROS are involved in the BK reduction of RPE monolayer resistance. Notably, vasoinhibins prevent BK actions in both endothelial and RPE monolayers.

Consistent with previous studies (Abdouh et al., 2003), we demonstrate that intravitreal injection of BK in rats stimulates Evans blue dye permeation, indicating increased permeability of the BRB. The dose of BK we used (10 μM) promotes vasodilation (Gonzalez et al., 2004), but given that BK binds to the B2 receptor with an EC_50_ of 2 nM (Gera et al., 2011), we assume that this dose is pharmacological. Nonetheless, high levels of BK may be found in the eye under conditions of diabetic retinopathy, when the levels of carbonic anhydrase I, an enzyme responsible for generating BK (Han et al., 2002; Gao et al., 2007), are ~15- and ~8-times higher than in non-diabetic patients and in patients with diabetes without retinopathy, respectively (Gao et al., 2007). Additionally, vasoinhibins opposed the BK action at concentrations previously demonstrated to prevent the vascular hyperpermeability observed in diabetes (Garcia et al., 2008). Earlier studies found that a B2 receptor antagonist reduces the retinal plasma extravasation induced by BK (Abdouh et al., 2008). Here, we show that Hoe-140, the competitive antagonist of B2 receptors, blocked the BK-induced increase in BRB transport, as did vasoinhibins. Because B1 receptors were not detected in the retina of rats subjected to BK treatment and because the competitive B2 receptor antagonist Hoe-140 fully blocked the BK effect on BRB permeability, we conclude that the BK-induced increase in BRB transport depends on B2 receptor stimulation. Furthermore, vasoinhibins have been shown to antagonize other B2 receptor-mediated effects of BK (Gonzalez et al., 2004; Thebault, 2011), the inhibition of phospholipase C activity being the most upstream target reported for vasoinhibins in the BK signaling pathway (Thebault, 2011). Given that changes in B2 receptor mRNA levels have been detected immediately after stress exposure, leading to changes in protein levels at 3 h (Nostramo et al., 2013), we postulated that vasoinhibins could down-regulate B2 receptor expression within the 2-h period during which we observed the *in vivo* effect of BK. However, there were no changes in B2 receptor mRNA and protein levels in the retina of rats subjected to BK treatment with or without vasoinhibins. Consequently, we propose that vasoinhibins negatively regulate B2 receptor signaling by short-term mechanisms that may include receptor desensitization (Mathis et al., 1996) and internalization (Munoz and Leeb-Lundberg, 1992; Munoz et al., 1993).

Stimulation of B2 receptors has been shown to evoke intracellular Ca^2+^ mobilization in cultured retinal capillary endothelial cells and to induce vascular permeability, vasodilation, and thickening of the retina, substantiating that the intraocular kallikrein-kinin system is functional in the retina (Abdouh et al., 2003). However, the only known BK effects on retinal non-vascular cells are to promote glutamate uptake by ARPE-19 cells (Lim et al., 2008) and to decrease retinal glutamate toxicity in cultured retinal neurons (Yasuyoshi et al., 2000). Our data provide the first demonstration that BK transiently reduces the trans-electrical resistance through ARPE-19 cell monolayers. This action shares similarities in amplitude and kinetics with the BK action on endothelial cell monolayers derived from freshly isolated retinal and brain capillary endothelial cells, and also from BUVEC, and it supports the role of BK as a direct regulator of both the inner endothelial and outer RPE components of the BRB. In addition to confirming that BK enhances transport through the brain-blood barrier (Harford-Wright et al., 2011), these findings extend BK actions to other blood-organ barriers.

Activation of the B2 receptor stimulates the NO production *via* Src/PI3kinase/Akt-dependent activation of eNOS (Bae et al., 2003), which has been associated with alterations in endothelial resistance and permeability (Pricci et al., 2003; Wilkinson-Berka, 2004; Frey and Antonetti, 2011). Our findings confirm that exogenous NO reduces the electrical resistance through endothelial monolayers and extend this effect of NO to the RPE. We also found that pharmacological inhibition of NO production leads to inhibition of the BK-induced increase of BRB transport and of permeability through both endothelial and RPE monolayers. Concerning the latter, even though constitutive NOS expression has not been described in ARPE-19 cells, RPE is positive for NADPH-diaphorase, a marker of NOS activity (Goldstein et al., 1996; Fischer and Stell, 1999). Our data also showed that vasoinhibins block the transient decrease in BUVEC and ARPE-19 monolayer resistance induced by BK, as did the NO synthase inhibitor L-NAME. In addition, the NO donor DETANONOate prevented vasoinhibins from blocking the BK effect in both cell types, supporting that the vasoinhibin act by inhibiting NO production, as previously demonstrated for endothelial cells (Gonzalez et al., 2004). While this is the first evidence that RPE cells respond to vasoinhibins, we previously demonstrated that vasoinhibins block BK-induced endothelial NO production by inactivating eNOS *via* two mechanisms: reduced intracellular Ca^2+^ mobilization (Gonzalez et al., 2004) and activation of protein phosphatase 2A-induced dephosphorylation at serine^1179^ (Garcia et al., 2008; Thebault, 2011).

NO can also stimulate protein kinase G, which causes the release of mitochondrial ROS into the cytosol (Oldenburg et al., 2004), and BK induces oxidative stress through an NO-independent mechanism in the human vasculature (Fong et al., 2010). However, neither endothelial nor RPE cells showed increased levels of ROS as byproducts during mitochondrial electron transport in response to BK, which may be due to NO-induced inhibition of ROS production (Kayashima et al., 2012). Indeed, NO produced in response to BK (Gonzalez et al., 2004) may impede ROS production by negatively regulating oxidative phosphorylation through the inhibition of cytochrome c oxidase. Also, BK did not modify the levels of superoxide formed as an intermediate of metal-catalyzed oxidation reactions. Moreover, we found that the radical initiator Luperox induced an increase in endothelial cell permeability with longer latency and duration compared to the effect of BK, and that the antioxidant NAC did not modify the increased permeability due to BK; thus, ROS may not contribute to the BK-induced decrease in endothelial cell resistance.

In contrast, our data indicate that ROS participate in the BK-mediated regulation of RPE resistance. Luperox mimicked the BK effect on ARPE-19 monolayer resistance in terms of amplitude, kinetics and actin cytoskeleton rearrangement, coinciding with the disruption of junctional proteins previously reported in association with increased ROS (Miranda et al., 2012; Zavodnik et al., 2013 and present data). BK *per se* caused superoxide anion, but not ROS, to accumulate in ARPE-19 cells as a by-product during mitochondrial electron transport. Consistent with these data, the antioxidant NAC prevented the decrease in ARPE-19 monolayer resistance induced by BK. Moreover, our data show that vasoinhibins mimicked the effect of NAC and prevented the transient reduction of ARPE-19 resistance induced by Luperox, indicating that vasohibins act by antagonizing ROS. Along this line, the intravitreal injection of vasoinhibins in the diabetic rats reduced the retinal levels of superoxide anion to control values. These data unveil vasoinhibins as potent anti-oxidants in the diabetic retina. We observed that a 45-min treatment with vasoinhibins diminished ROS levels in endothelial cells. Even though the same *in vitro* assay showed that vasoinhibins did not block the generation of ROS induced by Luperox, vasoinhibins did inhibit the BK-induced increase of superoxide anion levels in ARPE-19 cells, as did NAC. These findings support the idea that inhibition of ROS production contributes to the vasoinhibin inhibition of permeability. An additional mechanism may be that vasoinhibins promote anti-oxidant mechanisms, thereby protecting RPE against oxidant-mediated monolayer dysfunction (Ho et al., 2006) and endothelial cells from oxidant-mediated permeability dysregulation (Yamagishi et al., 2006; Sheikpranbabu et al., 2010; Kim et al., 2012) and damage (Banumathi et al., 2010; Dou et al., 2012). The pigment epithelium-derived factor (PEDF) acts in this way, and vasoinhibins may, therefore, share some molecular actions with PEDF. Indeed, both vasoinhibins and PEDF stabilize the actin cytoskeleton in both endothelial and RPE cells. Also, vasoinhibins may preserve the reduced/oxidized glutathione ratio (Miura and Roider, 2009) and maintain normal membrane occludin and N-cadherin structure by blocking the ROS-induced stress kinase p38/27-kDa heat shock protein signaling, which is known to mediate actin rearrangement (Ho et al., 2006). Interestingly, prolactin, the molecule from which vasoinhibins originate (Clapp et al., 2006), was recently shown to prevent the decrease in brain-blood barrier permeability induced by metamphetamine by maintaining the levels of claudin-5 and occludin (Rosas-Hernandez et al., 2013).

In conclusion, vasoinhibins prevent the actions of BK on transport through BRB and on the resistance of endothelium and RPE. Vasoinhibin action is NO-dependent in both endothelial and RPE cells and is ROS-dependent only in the RPE. Consistent with these actions, vasoinhibins reduced the levels of ROS in retinas of streptozotocin-diabetic rats, including the RPE. These data extend the functions of BK in the retina, consolidate our previous findings showing that vasoinhibins protect the BRB under diabetic conditions, and provide novel molecular and cellular insights into the mechanisms of vasoinhibin action in the retina. Ultimately, because preclinical studies have shown that the administration of several inhibitors of kinin receptors (Phipps et al., 2009; Clermont et al., 2011; Liu and Feener, 2013) and of vasoinhibins (Ramirez et al., 2011) to diabetic rats ameliorates retinal vascular hyperpermeability and inflammation, this study supports the use of vasoinhibins as therapeutic agents to minimize BRB dysfunction.

## Author contributions

Conceived and designed the experiments: David Arredondo Zamarripa, Carmen Clapp, and Stéphanie Thebault. Performed the experiments: David Arredondo Zamarripa, Nundehui Díaz-Lezama, Rodrigo Meléndez García, Jesús Chávez Balderas, Norma Adán, Maria G. Ledesma-Colunga, Edith Arnold, and Stéphanie Thebault. Analyzed the data: David Arredondo Zamarripa, Nundehui Díaz-Lezama, Rodrigo Meléndez García, and Stéphanie Thebault. Contributed reagents/materials/analysis tools: David Arredondo Zamarripa, Edith Arnold, Maria G. Ledesma-Colunga, Norma Adán, Carmen Clapp, and Stéphanie Thebault. Wrote the paper: David Arredondo Zamarripa, Carmen Clapp, and Stéphanie Thebault.

### Conflict of interest statement

The authors declare that the research was conducted in the absence of any commercial or financial relationships that could be construed as a potential conflict of interest.
